# Transcriptomic Analysis Reveals Possible Influences of ABA on Secondary Metabolism of Pigments, Flavonoids and Antioxidants in Tomato Fruit during Ripening

**DOI:** 10.1371/journal.pone.0129598

**Published:** 2015-06-08

**Authors:** Wangshu Mou, Dongdong Li, Zisheng Luo, Linchun Mao, Tiejin Ying

**Affiliations:** College of Biosystems Engineering and Food Science, Fuli Institute of Food Science, Zhejiang Key Laboratory for Agro-Food Processing, Zhejiang R & D Center for Food Technology and Equipment, Zhejiang University, Hangzhou, People’s Republic of China; University of Malaga-Consejo Superior de Investigaciones Científicas, SPAIN

## Abstract

Abscisic acid (ABA) has been proven to be involved in the regulation of climacteric fruit ripening, but a comprehensive investigation of its influence on ripening related processes is still lacking. By applying the next generation sequencing technology, we conducted a comparative analysis of the effects of exogenous ABA and NDGA (Nordihydroguaiaretic acid, an inhibitor of ABA biosynthesis) on tomato fruit ripening. The high throughput sequencing results showed that out of the 25728 genes expressed across all three samples, 10388 were identified as significantly differently expressed genes. Exogenous ABA was found to enhance the transcription of genes involved in pigments metabolism, including carotenoids biosynthesis and chlorophyll degradation, whereas NDGA treatment inhibited these processes. The results also revealed the crucial role of ABA in flavonoids synthesis and regulation of antioxidant system. Intriguingly, we also found that an inhibition of endogenous ABA significantly enhanced the transcriptional abundance of genes involved in photosynthesis. Our results highlighted the significance of ABA in regulating tomato ripening, which provided insight into the regulatory mechanism of fruit maturation and senescence process.

## Introduction

The plant hormone abscisic acid (ABA) has been considered to be mostly associated with regulation of non-climacteric fruit ripening in previous reports [1–4]. However, it has been confirmed that ABA is also able to influence the ripening process of climacteric fruit, such as tomato, banana, peach, mango and melon [5–9]. Mounting evidences suggested that exogenous ABA could accelerate fruit maturation with promotion of many ripening-related biological events. For instance, decreased fruit firmness was observed in ABA-treated mango fruit, which resulted from higher activities of enzymes involved in fruit softening [9]. Exogenous ABA was also found to affect tomato fruit pigmentation with carotenoids accumulation and chlorophyll degradation [10, 11]. Meanwhile, it has been proposed that high level of ABA would stimulate the generation of ROS, which subsequently induced many genes involved in the antioxidant defense system [12, 13]. Moreover, the increase of ABA could also enhance the activity of PAL and CHS (the full names for all abbreviations presented in the article can be referred in S19 Table, similarly hereinafter.), which were crucial for flavonoids accumulation [4, 14].

Despite the predominant regulatory role of ethylene, ABA also functions as a significant hormone in the control of climacteric fruit ripening [15]. In the early stage, the accumulation of ABA precedes ethylene production, and the ABA content reaches to a peak level before ripening in various kinds of tissues [6, 7, 16]. ABA may act as an original inducer for the initiation of ripening, by triggering the expression of many ethylene-independent genes [6, 7, 17]. Ethylene, however, may be more important in the regulation of later ripening stages [6, 7]. Besides, many studies have observed that some ripening related events were not fully regulated by ethylene, which also support that ABA might function as an upstream regulatory factor before ethylene in the ripening process [6, 7, 18–21]. Additionally, when the increasing ABA reaches to a certain level, it could in turn stimulate the expression of *ACS*s and *ACO*s, thus promoting the transformation of ACC to ethylene [22–24]. Therefore, exogenous application of ABA to fruit could induce ethylene biosynthesis and lead to a series of physiological and biological responses that facilitate fruit ripeness [7, 9, 20, 25]. The reduction of ABA level by NDGA or other inhibitors could result in delayed ripening, along with suppression of ethylene production in fruit [6, 7, 9, 20]. Taken together, these studies raised the hypothesis accounting for the biological effects of ABA on ripening processes, but a comparative analysis of the genetic evidences is still lacking.

As a typical climacteric plant, tomato is an ideal model for the study of fleshy fruit development and ripening. The available tomato genome data provides an advantage to perform genome-wide transcriptome analysis to study gene expression patterns under various conditions [26]. To comprehensively understand the mechanism of hormonal regulation on tomato fruit ripening, the next-generation RNA sequencing (RNA-seq) has been applied to detect all expressed genes at the transcriptome level [27, 28]. Shi et al. conducted a transcriptome analysis of cytokinin response in tomato leaves at different ages [29], the results showed that genes related to cytokinin signaling, metabolism and transport were remarkably induced by exogenous cytokinin treatment in young leaves [29]. In addition, a comparative transcriptome analysis of tomato leaves has been conducted to explore the genes expression with the treatment of exogenous ABA [30], and the data elucidated that exogenous ABA was able to activate the genes encoding the ABA signaling pathway elements and the key proteins involved in ethylene biosynthesis and signal transduction [30]. Besides, a large number of genes associated with the ROS scavenging system, pathogen resistance and heat shock proteins were also identified to be up-regulated in response to exogenous ABA [30]. During the last few years, RNA-seq analysis has revealed the role of plant hormones in defense response of tomato roots and leaves, but our understanding about the genome-wide profiling of genes in response to ABA during tomato fruit ripening remains unknown [31–33].

In the present study, we employed the Illumina RNA sequencing to perform a transcriptome analysis of ABA response in tomato fruits [30]. In order to double-check genes affected by ABA, we also set up a NDGA treatment group to evaluate genome-wide transcription changes when the endogenous ABA biosynthesis was inhibited. This study would provide valuable data for exploration of the molecular mechanism of ABA regulation in many ripening related secondary metabolism processes, including fruit color variation, antioxidants metabolism, flavonoids biosynthesis. In addition, based on the primary data about ripening-related events from RNA-seq, we could be provided more information to comprehensively analyze the crosstalk between ABA and other phytohormones in our future work.

## Materials and Methods

### Plant materials and treatments

Cherry tomatoes (*Lycopersicon esculentum var*. *cerasiforme* ‘XinTaiyang’) were planted in standard culture greenhouses (20–25°C, relative humidity (RH) 70%-85%) of Transfar Agriculture Co Ltd (Xiaoshan, Zhejiang, China). Fruits were harvested at mature green (MG) stage and transported to the laboratory immediately. 600 fruits with uniform size and free from blemishes were selected, and randomly divided into three groups. For each group, either 25 μL of ABA (10 mM) aqueous solution, or NDGA (1 mM) aqueous solution, or distilled water (control) was injected into each fruit from the pedicle with the same depth by microsyringe, respectively. The injection method and chemical concentration were chosen based on preliminary experiments. Fruits were stored at 20°C (90% RH) in the dark for 18 days. Samples of the 9^th^ day which showed the most observable differences in progress of ripening among three groups were chosen for RNA sequencing. For sampling, fruit calyx and seed were removed, and the pericarps were flash-frozen in liquid nitrogen and stored at -80°C before RNA extraction.

### Measurement of Fruit Color and Firmness

Ten fruits per replicate of each treatment were used to determine surface color and firmness, respectively. L*, a*, b* were recorded by a Chroma meter (KONICA MINOLTA, CR-400, Japan), of which, a* was used to depict fruit color variation. Fruits’ firmness was assessed with a digital texture analyzer (TA-XT2i, Stable Micro Systems Ltd.; Godalmin, UK) with a 5 mm diameter probe. The maximum force (N) was recorded during the fruit being penetrated 10 mm at a rate of 1mm/s, and four different points around the equator of the fruit were tested on each fruit. All analyses were conducted in three replicates.

### Pigments Quantification

The content of total carotenoids and chlorophyll was measured according to Zhu et al. with a slight modification [34]. Pericarp tissue (1 g) was extracted with 10 ml of 60: 40% (v/v) hexane: acetone at 4°C for 24 h in the dark and then centrifuged at 12000 × g 4°C for 20 min. The optical absorbance of supernatant was measured at 663, 647 and 450 nm against a hexane blank using a UV-VIS spectrophotometer (UV-1750, SHIMADZU, Japan). The total chlorophyll and carotenoids content were calculated with the following equations: total chlorophyll (mg ml^-1^) = 8.02 (OD_643_) + 20.2 (OD_647_) and total carotenoids content (mg ml^-1^) = (OD_450_)/0.25.

The assay of lycopene and β-carotene contents were conducted by HPLC based on the method of Ronen et al. with minor modifications [35]. Pericarp tissue (1 g) was homogenized with 10 mL cold acetone and incubated in the dark overnight. After centrifugation, the supernatant was collected and then filtered through a 0.45 μm filter. Carotenoids were separated by reverse-phase HPLC using a Zorbax SB-C18 column (silica 5 μm, 4.6 mm × 250 mm) (Agilent, USA). Sample of 20 μL was injected into a Shimadzo LC2012A pump (Shimadzo Corp., Tokyo, Japan). The mobile phase was consisted of acetonitrile: H_2_O (9: 1) (solvent A) and 100% ethyl acetate (solvent B), and was used in a linear gradient between A and B for 30 min with a flow of 1 ml min^-1^. Lycopene and β-carotene contents were detected at a wavelength of 475 nm. The external standard method was used for quantification.

### mRNA-seq Library Construction for Illumina Sequencing

Total RNA extraction, mRNA purification, and cDNA library construction were conducted by LC-BIO (Hangzhou, China). Total RNA samples were prepared using the Total RNA Purification Kit, TRK1001 (LC Science, Houston, TX), treated with RNase-free DNase I following the manufacturer's procedure to remove contaminated genomic DNA. For each group, total RNA was extracted from 10 randomly selected fruits. Then the quantity and purity of total RNA were analyzed with Bioanalyzer 2100 and RNA 6000 Nano LabChip Kit (Agilent, CA, USA). For each treatment sample, equal quantities of high-quality RNA from 10 fruits were pooled for cDNA library construction.

The poly (A) messenger RNA was isolated from the total RNA samples with oligo (dT) attached magnetic beads (Invitrogen). The mRNA was fragmented into short fragments using divalent cations under elevated temperature. The cleaved RNA fragments were reverse-transcribed to the first-strand cDNAs by random hexamer primers. Then the second-strand cDNAs were synthesized to construct the final cDNA library according to the instruction of mRNA-Seq sample preparation kit (Illumina, San Diego, USA). The paired-end cDNAs had a length of 300 bp (± 50 bp). After that, the cDNA libraries were sequenced on the Illumina HiSeq 2000 platform at the LC-BIO (Hangzhou, China) following the manufacturer’s recommendations. The raw sequencing data can be downloaded from NCBI Sequence Read Archive (SRA) under the accession number GSE63521.

### RNA-seq Reads Mapping

After removing the low quality reads (i.e. reads containing sequencing adapters; reads containing Ns (unknown sequence) > 5; nucleotide with q quality score lower than 20) from the raw sequencing data, the paired-end reads were aligned to the *S*. *lycopersicum* genome (ftp://ftp.jgi-psf.org/pub/compgen/phytozome/v9.0/Slycopersicum/assembly/) by using Tophat version 2.0.9 [36], allowing a maximum of two base mismatches and multiple alignments per read (up to 20 by default).

### Transcript Assembly and Abundance Estimation

The mapped reads were then assembled by Cufflinks version 2.1.1 for further annotation and measurement of relative abundances of transcripts [37]. Gene expression level within each sample were normalized with values for fragments per kilobase of exon per million fragments mapped (FPKM). Then Cuffmerge was applied to comerge all transcripts of the three samples to generate unique transcripts, and Cuffdiff reestimated the abundance of the transcripts and concurrently tested for different expression. The genes were considered as significantly differentially expressed between two groups if their absolute value of log_2_ fold change > 1 and P value ≤ 0.05. Besides, in order to identify DEGs probably regulated by ABA more comprehensively and objectively, we conducted the comparison of genes expression among three groups (ABA, NDGA and CK). In triadic analysis of differential expression, the genes with P value ≤ 0.05 were identified as the DEGs by the method of Chi-square test. Meanwhile, in the analysis of DEGs, our gene expression level (FPKM value) was normalized by the Z-score determined with the formula: *Z* = (*X- μ*) / σ before heat-map plotting, where *X* represents the FPKM of a gene in a specific sample/time point, and *μ* and *σ* are the mean transcript expression and standard deviation of a gene across all samples, respectively [38].

### Bioinformatics Analysis

We annotated each tomato gene with Gene Ontology (GO) terms for biological processes, molecular functions, and cellular components by Blast2GO vision 2.2.25 with default parameters (http://www.geneontology.org/) [39]. Meanwhile, the GO enrichment analysis was conducted by performing Fisher’s exact test with P value ≤ 0.05. The information of pathway for each tomato gene was attained from Kyoto Encyclopedia of Genes and Genomes (KEGG) database from the online KEGG Automatic Annotation Server (http://www.genome.jp/kegg/). The differentially expressed genes (DEGs) were also subjected to GO function and KEGG pathway enrichment analysis, and those genes with P-value ≤ 0.05 were defined as significantly enriched GO terms/KEGG pathways. Besides, each gene was aligned to the Cluster of orthologous groups for eukaryotic complete genomes (KOG) database to predict and classify protein functions.

### Validation of RNA-seq Results by Quantitative Real-time PCR (qRT-PCR)

In order to verify the transcriptome data, expression level of a randomly selected set of differentially regulated genes was measured by qRT-PCR. The total RNA was extracted from 10 fruits of each treatment by RNAiso Plus (TaKaRa, Japan) and then was pooled at equal quantity. After that, the RNA of three treatments (ABA, NDGA and CK) was reverse-transcribed to cDNA with RNA PCR kit (TaKaRa, Japan), respectively. qRT-PCR was performed with SYBR Premix Ex Taq (TaKaRa, Dalian, China) on ABI Step One RT-PCR system according to the manufacturer’s instructions. Three biological replicates were performed and the primer names and corresponding sequences were listed in S18 Table. Relative expression was normalized to the internal control gene β-actin gene with 2^-ΔΔCT^ method [40]. The untreated sample (CK) was set as the calibrator for relative expression level. Pearson’s correlation was performed to determine the correlation of genes expression in ABA and NDGA treatments relative to the control between qRT—PCR and sequencing.

### Statistical analysis

For all the biochemical measurements, data were analyzed using SPSS software and values were presented as mean ± standard error. The statistical significance of the differences between the samples was analyzed by the least significant difference test (LSD) at a significance level of P = 0.05. Significantly different values evaluated by t test for P value < 0.05 were indicated with asterisks.

## Results

### General Effects of ABA and Sample Preparation for RNA-seq

**Fig 1:**
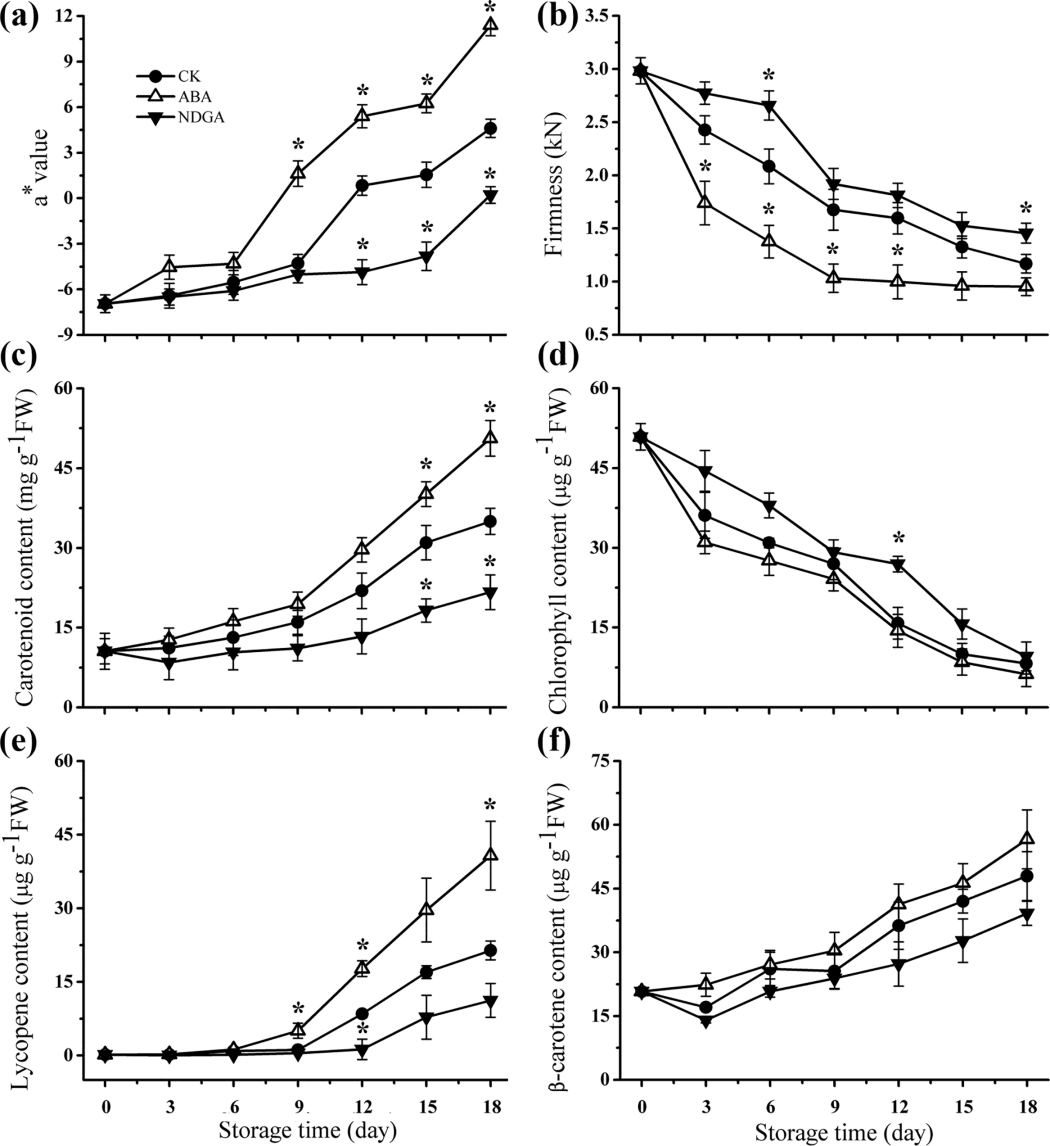
Effects of ABA and NDGA applications on tomato ripening-related physiological indexes during storage at 20°C. (a) The changes of a* value. (b) The changes of fruit firmness (kN). (c) The changes of total carotenoid content during tomato ripening. (d) The changes of chlorophyll content. (e) The changes of lycopene content. (f) The changes of β-carotenoid content. Error bars represented SE of three biological replicates, and asterisks (*) indicates significant difference (P<0.05) between the value in ABA treatment group or NDGA treatment group and that in CK (control).

To conduct transcriptome analysis of ABA influence on tomato fruit ripening, we selected the samples at specific ripening stage which exhibited the most considerable distinctions among different treatments for high throughput sequencing. During 18 days’ storage, the redness of tomato increased gradually, coinciding with the decline of fruit firmness in all samples (Fig 1a and 1b). In specific, total carotenoids increased as well as chlorophyll degraded during the fruit ripening (Fig 1c and 1d). Besides, lycopene and β-carotene, as the major carotenoids of tomato, accumulated progressively, which contributed to fruit color transition (Fig 1e and 1f). Compared with CK, ABA treatment remarkably promoted fruit ripening, whereas NDGA inhibited the process. Therefore, based on the morphological results, we chose the fruit samples on 9^th^ day to profile transcription changes via RNA-seq technology.

### Global Analysis of Fruit Transcriptome

The RNA samples from pericarps of ABA/NDGA/CK tomatoes were subjected for high-throughput sequencing performed on an Illumina HiSeq 2000 platform. After trimming low-quality reads, each cDNA library yield 43–51 million sequence reads, representing a total clean database of 14.37 Gb (S1 Table). The qualified sequence reads were mapped against the *S*. *lycopersicum* reference genome by TopHat with at most two mismatches tolerance. 3.29–3.82 million reads were mapped to the tomato genome, with an average of 74.81% of total reads aligned to the tomato genomic locations. In addition, 72.27% of CK, 70.41% of ABA and 71.83% of NDGA sequencing reads were uniquely mapped to the reference in each sample (S1 Table). After the assembly of all uniquely aligning reads, we obtained 34054 genes and 47933 transcripts which were identified in the transcriptome. Among those transcripts, 34580 (72.14%) matched completely with the annotated tomato genome, 6012 (12.54%) were unknown transcripts and 11606 (24.21%) were potentially novel isoforms (S2 Table).

### Function Classification and KEGG Analysis

Out of all 39690 genes, 11090 (27.94%) were successfully aligned to Gene Ontology (GO) and were classified into 41 functional groups, including 20 groups in biological process, 9 in cellular component and 12 in molecular function (S1a Fig). Within the class of cellular component, “cell” (GO:0005623) with 3254 genes and “cell part” (GO:0044464) with 3254 genes were predominant. In the category related to molecular function, a large amount of genes were involved in the “binding” (GO:0005488, 6190 genes) and “catalytic activity” (GO:0003824, 5521 genes). For the biological process, the assignments were mostly given to the terms of “metabolic process” (GO:0008152, 6022 genes) and “cellular process” (GO:0009987, 5142 genes) (S3 Table).

The total expressed genes were also blasted against the cluster of orthologous groups for eukaryotic complete genomes (KOG) of protein categories. A total of 10194 genes could be assigned to the KOG classification, and clustered into 25 functional categories. Among them, “signal transduction mechanism” (1316 genes, 12.91%) was found to be the major KOG category, followed by “general function prediction only” (1216 genes, 11.93%), “posttranslational modification, protein turnover, chaperones” (1107 genes, 10.86%) and “carbohydrate transport and metabolism” (618 genes, 6.06%). Additionally, only 577 (5.66%) genes were classified into the group of “function unknown” (S1b Fig).

All detected genes were subjected to KEGG pathway enrichment analysis, and 2403 genes were assigned to 144 pathways. The major pathways were “ribosome” (ko03010) (199, 8.28%), “oxidative phosphorylation” (ko00190) (110, 4.58%) and “spliceosome” (ko03040) (105, 4.37%), followed by “purine metabolism” (ko00230) (84, 3.50%), “glycolysis/gluconeogenesis” (ko00010) (82, 3.41%) and “plant-pathogen interaction” (ko04626) (74, 3.08%) (S1c Fig, S4 Table).

### Analysis of Differentially Expressed Genes

A total of 25728 genes (75.55% of 34054) were expressed in the tomato fruit transcriptome, and a Venn diagram was adopted to present the number of uniquely or commonly expressed genes in the three analyzed samples (S2a Fig, S5 Table). It was found that 18049 genes expressed commonly across all three samples. CK had 1270 unique genes, while ABA and NDGA had 1518 and 2191 specifically expressed genes, respectively. In addition, 843 common genes were identified in both CK and ABA, and 969 genes were detected in both CK and NDGA (S2a Fig).

In order to evaluate the regulatory role of ABA, differential expression analysis was performed between samples. The expression levels within a given sample were normalized with a value of FPKM (fragments per kilobase of exon per million fragments mapped). 44.68% of all genes were in 10–100 FPKM range, followed by those with expression level of 1–10 FPKM (35.15%), < 1 FPKM (11.7%) and 100–1000 FPKM (7.48%) (S6 Table). The genes with│log_2_ (fold change) ≥ 1 and P value ≤ 0.05 were identified as significantly differentially expressed genes in the comparison between two groups. Consequently, there were 10388 (40.38% of 25728) DEGs in total among the three samples (P value ≤ 0.05), and the number of DEGs in exogenous ABA or NDGA treated fruit was counted in S2b Fig.

We grouped all DEGs into GO functional categories in order to identify specific biological processes affected by exogenous ABA and NDGA. In the comparison between ABA and CK, proteins related to “photosynthesis, light harvesting” (GO:0009765, P value = 1.68E-06, regulation: down) were the most significantly enriched term in the biological process category, proteins associated with “photosystem I” (GO:0009522, P value = 7.70E-06, regulation: down) were highly enriched in cellular component and “ribulose- bisphosphate carboxylase activity” (GO:0016984, P value = 0.022, regulation: up, down) were the most highly represented in the category of molecular function. Meanwhile, the differentially expressed genes induced by NDGA were mainly distributed in the “photosynthesis, light harvesting” (GO:0009765, biological process, P value = 2.37E-05), “photosystem I” (GO:0009522, cellular component, P value = 3.50E-05) and “hydrolase activity, acting on ester bonds” (GO:0016788, molecular function, P value = 0.033) (S7, S8 and S9 Tables). These data suggest that the alteration of ABA may influence the expression of genes related to the GO function of “photosynthesis” in tomato fruit.

Next, the DEGs were further subjected to KEGG pathway enrichment analysis. It was found that “photosynthesis-antenna proteins” (ko00196) and “photosynthesis” (ko00195) were the most enriched pathways under the influence of exogenous ABA. The DEGs altered by exogenous NDGA were also involved in the similar KEGG pathways as observed in ABA, indicating a close relationship between photosynthesis and ABA (S10, S11 and S12 Tables).

### Analysis of Genes Related to Fruit Color Variation

**Fig 2:**
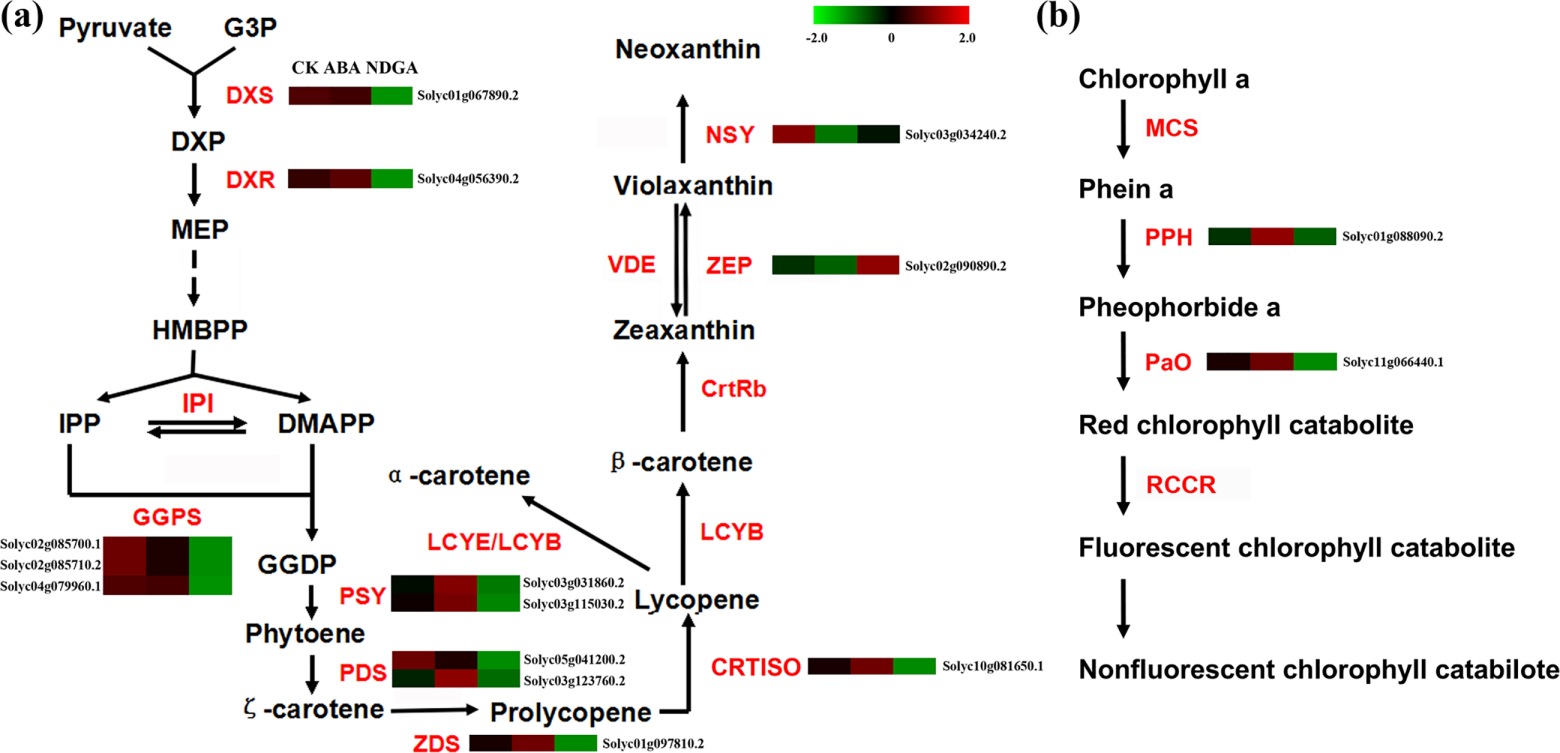
Analysis of DEGs involved in the pathway of carotenoid biosynthesis and chlorophyll degradation. (a) Schematic diagram of carotenoid biosynthesis (modified from the reference [78]) and the expression profile of 13 DEGs encoding key enzymes in the pathway. (b) Schematic diagram of chlorophyll degradation pathway (referred to reference [78]). The expression profile of 2 DEGs encoding key enzymes in chlorophyll degradation. Analysis of the transcriptional profiles of the relative gene expression values (Z scores) was performed using the heatmap command, Red and green color represent up-regulated and down-regulated genes, respectively. Abbreviations are listed in Supplemental S19 Table.

During tomato ripening, fruit color alters from green to red via the accumulation of carotenoids and a parallel reduction in chlorophyll concentration [41]. Lycopene and β-carotene are the major carotenoids in ripe tomato fruits [42, 43] (Fig 2a). Besides, chlorophyll is an extremely important biomolecule responsible for green color in the early stage of tomato ripening (Fig 2b) [44, 45]. In this study, 34 expressed genes were identified in color alteration process, with 13 DEGs in carotenoids biosynthesis and 2 DEGs in chlorophyll degradation, respectively (S13 and S14 Tables).

To further analyze effects of ABA or NDGA on carotenoids pathway, expression of genes encoding 12 key enzymes involved in the carotenoids biosynthesis were analyzed. DXS is regarded as the rate-limiting enzyme in the MEP (2-C-methyl-D-erythritol 4-phosphate) pathway. We detected two *DXS* genes expressed in the tomato transcriptome, and one of them was significantly down-regulated by NDGA (Solyc01g067890.2). Compared with CK, the expressed *IPI* was significantly up-regulated by ABA and down-regulated by NDGA (Solyc04g056390.2). The three expressed *GGPS*s were all identified as significantly changed, which were specially repressed by NDGA from 1.28 fold to 2.27 fold (Solyc02g085700.1, Solyc02g085710.2 and Solyc04g079960.1). PSY is regarded as a key component in the upstream of carotenoids pathway, which participates in the conversion from GGPP to phytoene. Among the three expressed *PSY*s, two of them showed obvious increase in expression level after ABA treatment as well as great reduction after NDGA treatment (Solyc03g031860.2, 2277.9 FPKM in CK, 2951.4 FPKM in ABA and 1768.0 in NDGA; Solyc03g115030.2, 204.8 in CK, 232.2 in ABA and 165.9 in NDGA). Some Other important enzymes, PDS, ZDS and CRTISO, which contribute to the production of lycopene were all significantly up-regulated by ABA and evidently suppressed by NDGA in their transcript abundance. However, our data revealed that the transcription of *ZEP* was repressed by ABA treatment and the enhanced by NDGAtreament (Solyc02g090890.2, 18.0 in CK, 11.2 in ABA and 44.4 FPKM in NDGA).

Chlorophyll degradation is recognized as a pivotal process in fruit degreening [44, 45]. We analyzed major genes involved in chlorophyll breakdown including: *Chlase*, *PPH*, *PaO* and *RCCR* (S14 Table). Two *Chlase* genes (Solyc06g053980.2 and Solyc12g005300.1) showed no differences at expression level in ABA or NDGA treatment. One of the expressed *PPH*s (Solyc01g088090.2) was identified as DEG, which showed increased transcript abundance from 45.8 to 64.1 FPKM upon treatment with ABA. Among three detected *PaO*s, one of them was elevated by ABA and remarkably suppressed by NDGA (Solyc11g066440.1, 62.3 FPKM in CK, 67.6 in ABA and 51.7 FPKM in NDGA).

### Genes Related to Flavonoid Biosynthesis

**Fig 3:**
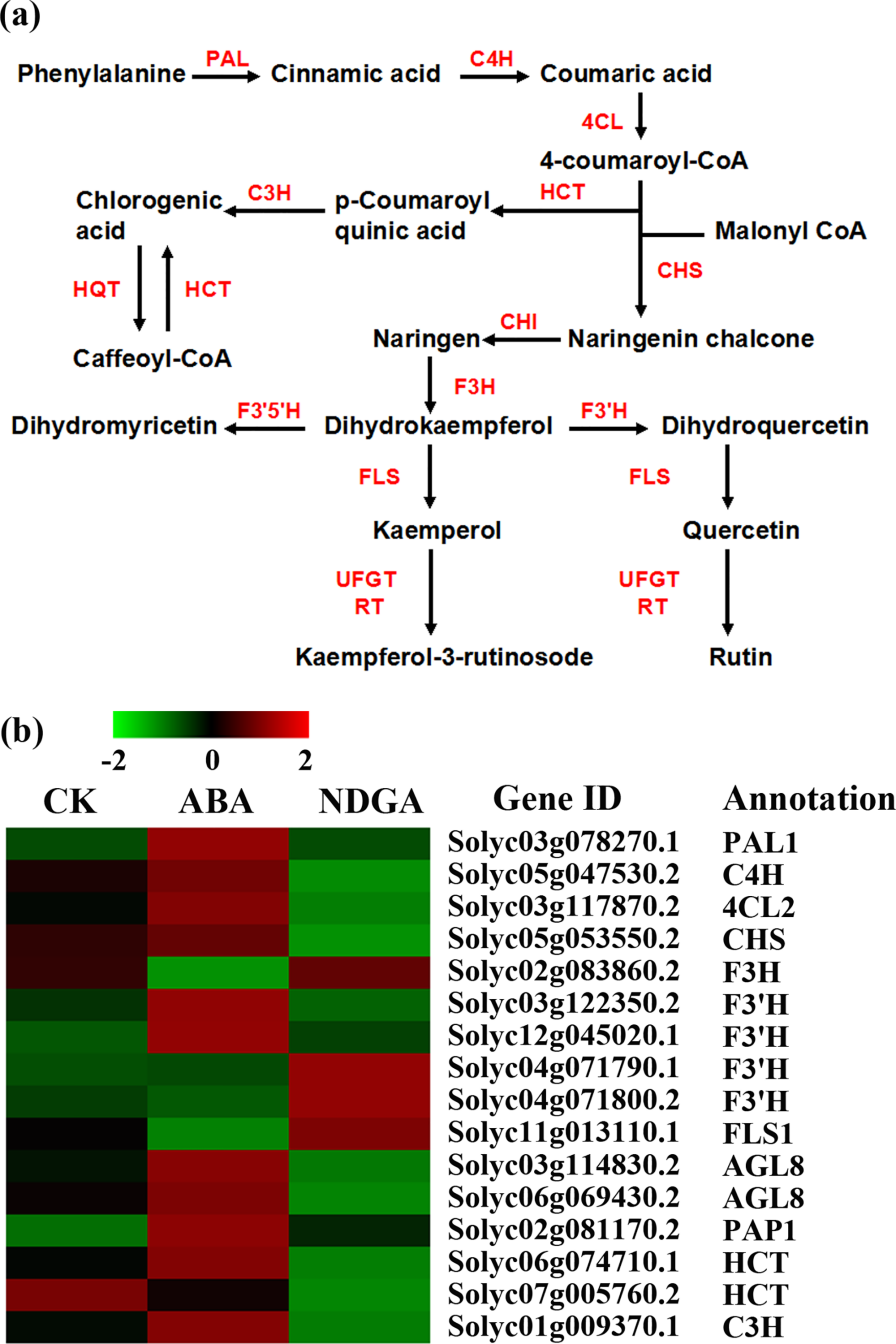
Analysis of DEGs involved in the pathway of flavonoids biosynthesis. (a) Schematic diagram of flavonoids biosynthesis (modified from the reference [49]). (b) The expression profile of 16 DEGs encoding key enzymes in the pathway. Abbreviations are listed in Supplemental S19 Table.

Flavonoids are ubiquitous plant secondary metabolites with a vast array of biological functions, including coloring, defense against biotic and abiotic stresses and contribution to plant growth and development [46]. Flavonoids are derived from the phenylpropanoid pathway, transforming phenylalanine into 4-coumaroyl-CoA, which demands the involvement of several crucial enzymes like PAL, C4H, and 4CL [47] (Fig 3a). There was only one *PAL* (Solyc03g078270.1) significantly regulated by exogenous treatments, which exclusively expressed in ABA treated-fruits (not expressed in CK and NDGA treatments) (Fig 3b). Transcription of the *C4H* (Solyc05g047530.2) was moderately improved by ABA from 24.7 to 33.6 FPKM, and repressed considerably to 7.7 FPKM by NDGA (Fig 3b). Moreover, of five *4CL*s, one with high expression level (Solyc03g117870.2) was up-regulated by ABA from 143.2 to 184.9 FPKM, and reduced to 105.7 FPKM by NDGA (S15 Table).

In tomato fruit, flavonoid naringenin chalcone (NarCh) has been reported as a predominant compound of total flavonoids, which was the first intermediate in the biosynthesis of flavonols [48]. It was synthesized from 4-coumarate-CoA by the catalysis of CHS and subsequently converted to naringenin (Nar) with the action of CHI [49]. In our result, the *CHS* gene (Solyc05g053550.2) expressed significantly higher in ABA treatment and showed a lower abundance in NDGA treated fruits (Fig 3b). However, none of *CHI*s expressed significantly differentially by either of the exogenous treatments. The expression of *F3H* was significantly decreased in ABA treatment by almost 2 fold (Solyc02g083860.2, from 130.3 to 67.4 FPKM), but slightly increased in NDGA treatment to 146.2 FPKM. The formed dihydroquercetin and dihydrokaempferol would be converted to kaempferol and quercetin respectively, which were catalyzed by the enzyme of FLS. It was observed that the *FLS* was suppressed by 1.51 fold in ABA treatment and induced by 1.31 fold in NDGA treatment (Solyc11g013110.1, 714.1 FPKM in CK, 474.8 FPKM in ABA and 932.5 in NDGA).

Many transcription factors have been previously proven to regulate the expression of flavonoids-pathway genes, such as *MYB12*, *AGL8* and *PAP1* [48, 50, 51]. We found that the two *AGL8*s both showed obviously increased expression when treated with exogenous ABA and correspondingly decreased upon NDGA application (Solyc03g114830.2, 165.6 in CK, 203.4 in ABA and 134.0 FPKM in NDGA; Solyc06g069430.2, 625.0 in CK, 772.0 in ABA and 445.7 FPKM in NDGA). Meanwhile, the transcript of *PAP1* (Solyc02g081170.2) was also enhanced by ABA, which increased from 174.7 to 203.9 FPKM.

### Genes Related to the Reactive Oxygen Species (ROS) Scavenging Enzymes

During fruit maturation, continuously generation of reactive oxygen species (ROS) could cause oxidative damages, initiating a variety of ROS-related disorders or cellular toxicity in plant [52]. ROS include free radicals like superoxide anion (O_2_^-^), hydroxyl radical (OH^-^) and other non-radical molecules such as hydrogen peroxide (H_2_O_2_), singlet oxygen (^1^O_2_) [52]. The formation of ROS can be scavenged by a battery of enzymes in the antioxidant system, which was mainly composed of the GSH-ASA cycle (32 detected genes), GPX pathway (99 detected genes), the PrxR/TrX pathway (74 detected genes), SOD (7 detected genes) and the CATs (6 detected genes) [30] (S16 Table).

**Fig 4:**
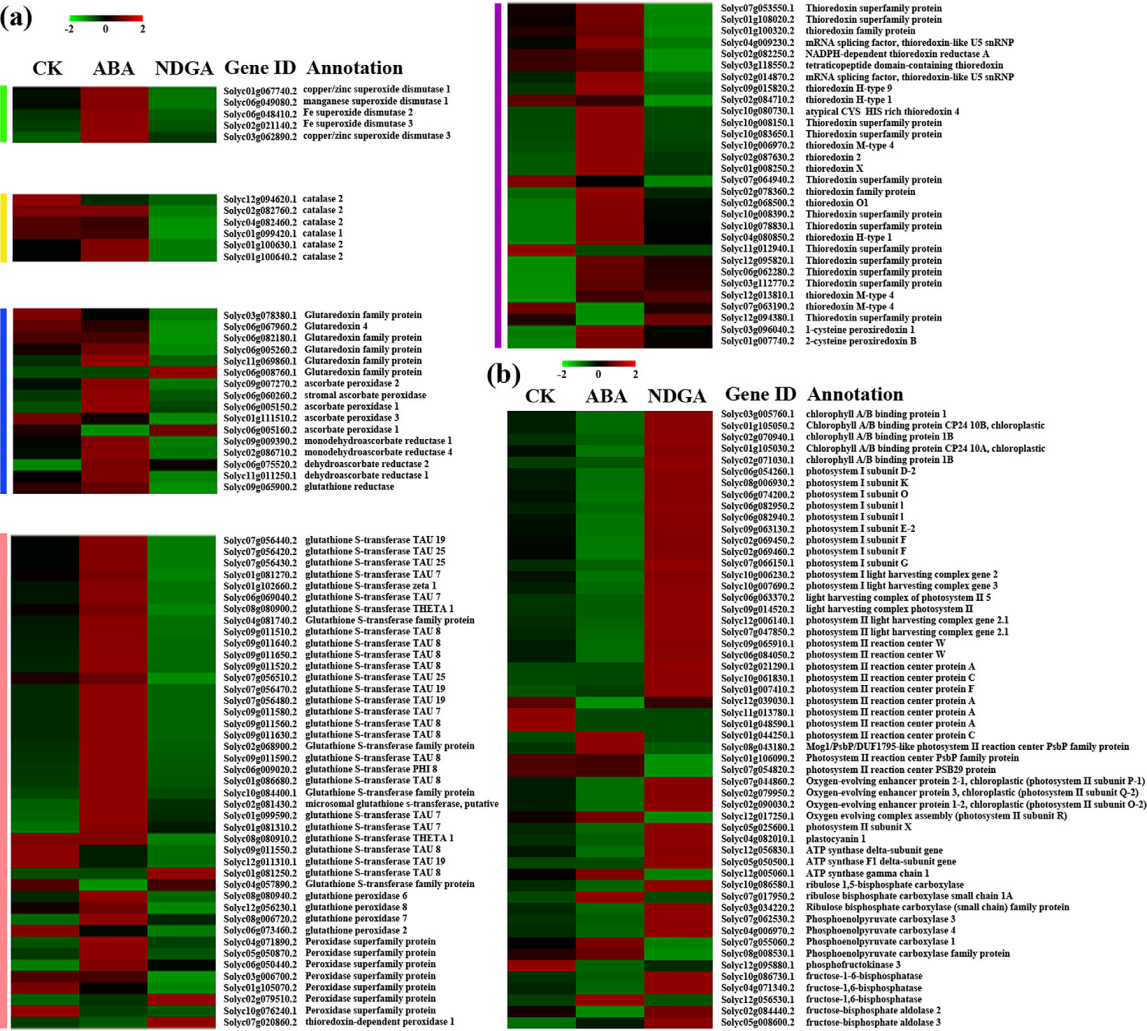
Analysis of DEGs involved in the pathway of ROS-scavenging and photosynthesis, respectively. (a) Expression profiling of DEGs involved in antioxidant system. Green bar, *SOD*s; yellow bar, *CAT*s; blue bar, genes encoding enzymes of GSH-ASA cycle; pink bar, genes encoding enzymes of GPX pathway; purple bar, genes encoding enzymes of PrxR/TrX pathway. (b) Expression profiling of DEGs encoding key enzymes in different steps of photosynthesis.

SODs were antioxidant defense enzymes, catalyzing the dismutation of superoxide radicals (O_2_^-^) into oxygen and H_2_O_2_ [53]. Of all seven *SOD*s, five were identified as DEGs, showing remarkably increased expression levels in ABA and decreased levels in NDGA treated samples (Fig 4a). CATs, similar to SOD, were also of great importance to keep the primary products of partial oxygen reduction at steady-state concentrations in cells and tissues [54]. Six expressed *CAT*s were all identified as DEGs, which showed slight alterations upon ABA treatment but noteworthy reductions by NDGA (Fig 4a).

In addition, the GSH-ASA cycle has been proved to be involved in the action of eliminating ROS, which can be stimulated by environmental stress [55]. ASA and GSH were the important elements of GSH-ASA cycle, and the ratios of AsA/DHA (dehydroascorbate) and GSH/GSSG (glutathione disulfide) were considered as signals for modulating antioxidant mechanisms [56]. There were 15 *GLR*s, 10 *APX*s, 3 *MDAR*s, 3*DHAR*s and 1 *GR* in the GSH-ASA cycle. *GLR*s are small disulphide oxidoreductases with catalytic activity of S-glutathionylated proteins, thus becoming key enzymes in redox signaling and ROS scavenging [57, 58]. Among 15 *GLR*s, there were 5 genes considered to be altered significantly after exogenous ABA or NDGA applications. The abundances of Solyc06g067960.2, Solyc03g078380.1, Solyc06g005260.2, and Solyc06g082180.1 in NDGA treatment were lower than those in control by 1.39, 1.32, 1.27 and 3.97 fold respectively. APXs were currently reported as the major peroxidase for reducing cellular H_2_O_2_ into water, by using ASA as electron donor [59]. Almost all *APX* DEGs exhibited considerably increased abundance with exogenous ABA but were little influenced by NDGA treatment (Fig 4a). Among three *MDAR*s, two of them (Solyc09g009390.2 and Solyc02g086710.2) were both significantly induced by ABA and repressed by NDGA treatment. Moreover, DHARs are also essential enzymes for reducing DHA back to AsA using GSH as reducing substrate [60]. Two *DHAR*s (Solyc06g075520.2 and Solyc11g011250.1) were significantly up-regulated by ABA and down-regulated by NDGA, and the only one detected *GR* (Solyc09g065900.2), which catalyzed the reduction of GSSG to GSH in conjuction with NADPH, also showed a significantly elevated expression in ABA and a reduction in NDGA samples.

The GPX pathway contained 51*GST*s, 6 *GPX*s and 42 *POD*s. GSTs are a superfamily of proteins, and there were 31 *GST*s showing different expressions upon exogenous ABA or NDGA treatment, most of which were significantly up-regulated by ABA and down-regulated by NDGA (Fig 4a). GPXs also have the catalysis activity of reducing H_2_O_2_ to water, by using GSH as the reductant [61]. Four *GPX*s were identified as DEGs, which also showed a similar changing trend like other genes. Eight DEGs were identified among the large family of *POD*s, and the majority of them were highly expressed in ABA treated fruits.

It has been proposed that TrX/PrX system has a vital role in the removal of H_2_O_2_ [62, 63]. It has been reported that the Trxs were central players of various signaling cascades including redox homeostasis, and the catalytic activity of oxidized PrxRs could be restored by Trxs [64]. A total of 70 *Trx*s were expressed in our samples, and almost all of the 29 DEGs were up-regulated by ABA. *Prx*s are a ubiquitous family of antioxidant enzymes, reducing peroxides by redox-active cysteines [65]. There were four genes encoding PrxRs in the present study, and three of them were in significantly higher abundance in ABA treatment than those in CK (Fig 4a).

### Genes Related to Photosynthesis Process

Interestingly, we observed that the GO term and KEGG pathway related to photosynthesis was highly enriched with the treatments of ABA and NDGA, indicating that the photosynthetic system and its components are regulated by phytohormone ABA during postharvest fruit ripening. There was a substantial amount of DEGs associated with different steps of photosynthesis, which showed significantly up-regulation in NDGA-treated fruits and down-regulation in ABA (Fig 4b, S17 Table). The vast majority of DEGs involved in photosystem I and photosystem II had remarkable higher expressions with NDGA application and decreased expressions in ABA treatment. Among 13 genes encoding chlorophyll A/B binding proteins, five of them showed significant differences in expression level. Regarding to photosystem I subunit, 9 DEGs were detected in the tomato transcriptome, all of them were up-regulated by NDGA and down-regulated by ABA. Two of *LHCA*s were identified as markedly changed in exogenous treatments. In particular, the expression of Solyc10g006230.2 increased from 63.2 to 160.9 FPKM after NDGA application, and dropped to 4.0 FPKM upon ABA treatment. All of four *LHCB*s were detected as DEGs in our analysis, which also showed the similar expression pattern with exogenous applications. There were 31 genes which encoded the photosystem II reaction center proteins expressed in this study. Of them, only two genes were significantly higher in NDGA and lower in ABA than control. Moreover, most of the genes involved in the generation of oxygen-evolving complex proteins and photosystem II subunit X were found to be induced in NDGA and repressed in ABA treatment as well (S17 Table). Besides, widespread up-regulations by NDGA were also observed in other genes related to light-dependent reactions, such as cytochrome b6f complex, plastocyanins, ATP synthases and NADP reductase (S17 Table).

Apart from that, a large number of DEGs were involved in Calvin cycle and other light-independent reactions of tomato fruit [66]. Notably, RUBPCO, which was one of key enzymes in CO_2_ fixation, was strongly activated by NDGA at transcript abundance (32.8 FPKM in CK, 22.0 in ABA and 66.8 in NDGA). In addition, expression of a gene encoding RBCB (Solyc03g034220.2) was significantly increased by 3.64 fold in NDGA treatment and declined by 10.00 fold in ABA treatment. PEPC functions in the formation of oxaloacetate, which was also an important part of Calvin cycle [67]. There are four *PEPC*s identified as DEGs in this study, and two of them (Solyc07g062530.2 and Solyc04g006970.2) exhibited increased expressions by NDGA along with decreased expressions by ABA. We also observed up-regulation of two *FBP*s and one *FBA* in NDGA treatment, the genes participating in light-independent actions.

### Real-time Reverse Transcription-PCR (RT-PCR) Validation of Selected DEGs

To validate the gene expression data from RNA-seq, 16 genes having different expression patterns were selected for real-time RT-PCR. Compared with the CK, the expression of all tested genes in ABA and NDGA treatments had a similar tendency between deep sequencing and qRT—PCR, respectively (S3 Fig, S18 Table). Besides, scatterplots by comparing the log2 fold change determined by RNA-seq and RT-PCR were also adopted in our study to confirm the accuracy of the transcriptomic results. Pearson’s correlation test showed that expression of the 16 genes relative to the control exhibited positive correlations between RNA sequencing and qRT—PCR (R^2^ = 0.86 in CK vs ABA; R^2^ = 0.70 in CK vs NDGA), indicating the high reliability of RNA-seq data of tomato transcriptome (S4 Fig). As for the difference of fold change between RNA-seq and qRT-PCR results, it may be caused by the different sensitivity of two different technologies [68]. In addition, the higher fold change observed in some genes by RT-PCR may be due to the exponential amplification in PCR that may boost real mRNA expression [68].

## Discussion

Although a number of studies have been performed to investigate the influence of ABA on tomato ripening, the genetic mechanism of ABA regulation in relevant ripening processes remain to be further elucidated. In the present study, we extended the fundamental understanding on the response of tomato fruits to ABA by comparing the global transcriptome changes among ABA-treated, NDGA-treated and the control samples. In particular, the DEGs involved in the pathways of carotenoids biosynthesis, chlorophyll metabolism, ROS scavenging system, flavonoids biosynthesis and photosynthesis were analyzed.

In previous studies, many kinds of ABA-deficient mutants (e.g. *hp3*, *flacca* and *sitiens*) which block ABA biosynthesis by the impairment of ZEP, MoCo or AAO respectively, showed the phenotypes of higher carotenoids content than wild type [15, 69]. The reduced ABA of these mutants may act as a stress signal to induce plastid division, thus leading to enlargement the total size of plastid compartment to store more carotenoids [15, 70]. In fact, the expression level of genes encoding rate-limiting enzymes PSY, DXS and GGPS did not show any significant changes, indicating that the enhanced carotenoids in ABA deficient mutants was not due to the up-regulation of key genes expression [15]. Apart from these natural mutants, the RNAi-mediated suppression of key enzyme NCED also lead to an obstruction for ABA biosynthesis, which could increase the carotenoids accumulation in the transgenic tomato [71]. The explanation for this phenotype could be that the reduced activity of NCED may cause the inhibition of carbon flux to ABA biosynthesis, so the ‘back-logged’ carbon transformed into upstream carotenoids production by up-regulation of *PSY*, *PDS*, *GGPS* and *LCYB* transcription in the RNAi lines [71].

However, many studies have reported that exogenous ABA could up-regulate the expression level of many key genes in the pathway of carotenoids biosynthesis and promote carotenoids accumulation [72–75]. Our study results (Figs 1 and 2a, S13 Table) added new evidence to support the positive relation between ABA and carotenoids biosynthesis. With respect to the discrepancy with the natural mutants or transgenic tomatoes (mentioned above), we assumed that the different phenotypes caused by ABA alterations may be regulated by different mechanisms. As we all know, carotenoids concentration would increase dramatically during fruit ripening, and the genes for carotenoids biosynthesis enzymes were also significantly up-regulated in the process of ripeness [11, 35, 76]. In our experiment, the high level of ABA by exogenous ABA application could induce ethylene synthesis (data not shown) and advance the onset of ripening [6, 7], thus accelerating carotenoid accumulation (Fig 1) and up-regulating key genes in contrast with control fruits (S13 Table). Meanwhile, different from the inhibition pattern of ABA in natural mutants or RNAi lines (mentioned above), NDGA application may consistently keep ABA content at a quite low level, which was insufficient to initiate fruit ripening [6, 7]. Therefore, it could be assumed that the lower level of carotenoids in NDGA treatment was attributed to the delayed ripeness (Fig 1). In our data, the transcriptional abundance of *DXS*, *IPI* and *GGPS* were significantly repressed by NDGA, implying the endogenous ABA may stimulate the initiation of carotenoid biosynthesis by regulating the expression of these genes [73]. As the crucial genes for carotenogenesis, the induced transcriptional level of *PSY*s, *PDS*s, *ZDS* and *CRTISO* may resulted in a remarkable increase of carotenoids content in ABA treatment and the reverse when NDGA was applied, which were in consistent with many other reports [77, 78]. Meanwhile, Dibari et al. have pointed out that the promoter of *PSY* gene would function in response to ABA, possibly leading to a marked transcriptional rise [77]. However, none of *LYB*s were significantly modified by ABA or NDGA in transcriptional abundance (S13 Table). This may be the reason why there was no significant difference observed in β-carotene content among CK, ABA and NDGA samples (Fig 1f). Besides, since the expression of *LYB*s weren’t significantly altered by ABA/NDGA, the accumulation of lycopene in three different samples (CK, ABA and NDGA) may be mostly influenced by the key elements in the upstream of lycopene, such as *PSY*s, *PDS*s, *ZDS* and *CRTISO* (Fig 2a). Unexpectedly, ZEP, which functions in the conversion of zeaxanthin to violaxanthin [79], was dramatically induced by NDGA at transcript abundance and slightly down-regulated by exogenous ABA (S13 Table). Despite no direct evidence to explain this interesting result, we presumed that the inhibition of endogenous ABA by NDGA might markedly induce the transcription of ZEP in order to produce more ABA to keep phytohormone balance in plants, which, however, requires to be further experimentally validated in the near feature.

In all, the influence of exogenous ABA or NDGA treatments on the carotenoids accumulation in our study was possibly resulted from the regulation of fruit ripeness, and the accelerated/delayed onset of ripening by ABA/NDGA may affect the gene transcription of key elements in carotenoids biosynthesis pathway, respectively.

Chlorophyll a was the major contributor to the total chlorophyll concentration, whose degradation accelerates fruit degreening (Fig 2b) [80]. In this study, exogenous NDGA treatment delayed the chlorophyll breakdown, whereas ABA promoted color transition (Fig 1d), agreeing with the results of many studies [80–82]. In our analysis, the detected *Chlase*s was not differentially expressed in all treatments, which was probably because chlorophyllases were not essential for senescence-related chlorophyll breakdown [83]. As many researches previously confirmed, PPH specifically dephytylated pheophytin and was indispensible in chlorophyll breakdown [84, 85]. In our data, one of *PPH*s was remarkably increased by ABA and repressed by NDGA in expression level, suggesting that PPH might play a major role in the promotion of the chlorophyll degradation by exogenous ABA. Other than PPH, PaO was also considered as a crucial enzyme in fruit degreening [84], and it’s transcription was also markedly induced/repressed by ABA/NDGA. Consistent with many previous researches, our result suggested that the high level of ABA could induce degreening process, possibly via enhancing expression of key enzymes involved in the chlorophyll degradation [86–89].

Analysis of flavonoid biosynthesis in tomato fruits showed that nine crucial enzymes and several transcription factors were involved in the metabolism of bioactive compounds which lead to the formation of NarCh, rutin and kaempferol-3-rutinoside (S15 Table). When the fruits were exposed to exogenous ABA, most of the genes encoding these important proteins were observed to be highly expressed except *F3H* and *FLS*. Consistent with many studies’ results, the significantly elevated transcription of *PAL*, *C4H* and *4CL* indicated the positive regulatory role of ABA in the production of 4-coumaroyl-CoA, which acted as the pivotal precursor for flavonoids biosynthesis [89–92]. It is reported that the strong activation of some transcription factors by ABA treatment promotes the expression of target genes encoding biosynthetic enzymes in phenylpropanoid pathway [89, 93]. In our data, the expression level of *CHS* was observed significantly regulated by ABA, demonstrating its predominant function as the first enzyme specific for the flavonoid accumulation [4]. However, the transcript levels of *CHI*s were observed relatively low, and most of them were slightly up-regulated by NDGA and suppressed by ABA. Similarly, it has been reported that CHI acted as a rate-determining enzyme in the formation of flavonols in tomato, whose mRNA levels remain low and even decrease upon ripening [49, 94]. Indeed, the decreased expression of *CHI* in ABA-treated tomatoes might explain the accumulation of its substrate, NarCh. Likewise, the significant down-regulation of F3H by ABA could also protect NarCh from transformation. FLS was also a key enzyme engaged in diverging point into the flavonol subclass branch, and its transcription was negatively regulated by ABA. This result was in agreement with the results of Li et al. [95]. Besides, AGL8 and PAP1, known as transcription factors, were both positively regulated by ABA content at transcriptional level, which may contribute to the flavonoids biosynthesis in fruit [51, 96]

ROS are generated as products of aerobic metabolism, which can cause an array of deleterious oxidations of cellular components. In order to protect cells from the toxic damages, plants can remove ROS by enzymatic antioxidant mechanisms [97]. It has been well accepted that the activity of many antioxidant enzymes would significantly increase during ripening, including APX, GR, CAT, SOD and MDAR, which act as defense response towards oxidative stress [98–100].

Regarding SOD, it has been considered as the crucial enzyme in regulating the level of O_2_^-^ and H_2_O_2_ [101]. Our analysis has shown that the *SOD* can be up-regulated by exogenous ABA and little affected by the reduction of endogenous ABA (Fig 4a), which was in accordance with other studies [13, 102, 103]. Guan et al. has observed that the expression of *CAT* was independent of endogenous ABA level, but could be enhanced by exogenous ABA treatment in maize [104]. Nevertheless, our data showed that all *CAT*s were suppressed following the decline of endogenous ABA, but showed minor alterations by exogenous ABA application. Besides, previous studies have showed that the activity of CAT stayed constant or even decreased in ABA-treated plant [12, 13, 102]. Therefore, these findings suggested that the regulatory mechanism of *CAT* by ABA treatment might vary in different plants. In GSH-AsA cycle, the genes of key elements were most significantly changed by exogenous ABA/NDGA treatments (Fig 4a). The marked repression of *GLR*s in NDGA treatment implied the important regulatory role of endogenous ABA. *APX*s, which emerges as central peroxidase for H_2_O_2_ reduction, was observed to be activated in ABA treated fruits but little affected by NDGA, supporting the idea that high ABA could contribute to a higher antioxidant capacity of plant by enhancing the production of APX [13, 59, 103–105]. Besides, our results also showed that the differentially expressed genes encoding *MDAR*s, *DHAR*s, *GR* and *GPX*s were improved by ABA and depressed when the endogenous ABA was inhibited, which was in accordance with the results of many studies [12, 105–107]. What’s more, *GPX*s have been demonstrated to interact with the negative regulators of ABA signaling (PP2Cs), also implying a link existed between ABA and GPX [61]. Furthermore, almost all DEGs of *Trx*s and *PrxR*s were up-regulated by ABA, which showed that the PrxR-Trx pathway was regulated by ABA at transcription level as well. In addition, it has been well known that ABA treatment can generate abundance of ROS, which in turn triggered the mitogen-activated protein kinase (MAPK) cascade and thus lead to increased expressions of antioxidant genes [12, 13, 107, 108]. However, the serious oxidative stress induced by ABA cannot be fully controlled in spite of the enhancement of whole antioxidant defense system, which finally aggravated the senesence of ABA-treated fruits [106].

It has been well established that the chlorophyll content in tomato fruit is retained up to a very advanced stage of ripening, and significantly decreases with the maturation of tomato fruit [66, 67]. The chloroplasts in fruit tissue clearly undergo a physiological transition into photosynthetically inactive chromoplasts, which may behave as respiratory organelles during ripening [109–111]. Therefore, the presence of Chl in green tomato, which functions as a consistent photosynthetic structure, indicates a capacity of photosynthesis in fruit [66–67, 112–115]. As fruit ripen, the degradation of chlorophyll (or remodeling into chromoplasts) appears to be coupled with a decline in the gene expression [116–119] and enzymatic activities [120–121] that are related to photosynthesis. Our transcriptomic data showed that the expression of genes encoding proteins of photosynthesis was significantly regulated by exogenous ABA and NDGA applications (Fig 4b). In specific, the tomato fruits with NDGA treatment sampled on the 9^th^ day demonstrated a conspicuous higher expression of genes encoding light-harvesting photosynthetic proteins, including reaction centers, photosystem I and photosystem II, and they were concomitant with the up-regulation of ATP synthase and NADP reductase. In addition, the genes associated with Calvin cycle also presented higher expressions when treated with NDGA. Correspondingly, the expression of most genes related to photosynthesis was repressed by ABA treatment (S17 Table, Fig 4b). The similar transcriptomic results also can be found in the grape berries upon ABA treatment [122]. Koyama et al (2010) hold the view that ABA could advance the switch from a photosynthetically active status to a sink status by accelerating fruit ripening, which resulted in the decreased abundance of many transcripts associated with photosynthesis [122, 123]. As previous studies demonstrated, the expression of photosynthesis genes is developmentally regulated [110]. Therefore, the significantly differential expression of photosynthetic genes observed in tomatoes may be a significant constituent part of regulation of fruit ripening by ABA. To date, our knowledge about the photosynthesis in postharvest fruits remains fragmentary, and little attention has been paid to the regulatory mechanism of fruit photosynthesis by ABA at transcriptomic level. So presenting our data about the effect of ABA on photosynthesis may be important to stimulate more studies on comprehensive proteome and transcriptome changes for a new insight into the interactions between photosynthetic system and postharvest fruit ripening.

In conclusion, the effect of exogenous ABA/NDGA applications on secondary metabolism pathways mentioned above may reveal a general scenario of fruit ripening regulated by ABA [6, 7]. Therefore, combined with the current knowledge of ripening regulation by other phytohormones, the sequencing data related to biological events may possibly provide us with a more in-depth understanding on multi-phytohormone regulation in fruit ripeining.

## Conclusions

Overall, next-generation sequencing enabled us to characterize the transcriptomic changes of tomato fruit treated with ABA and NDGA. By comparing these transcriptomes with control respectively, we observed that ABA could accelerate tomato fruit maturation by positively regulating many genes related to several important aspects of ripening process. Our study have turned spotlight on the pathways of fruit pigmentation, including carotenoids biosynthesis and chlorophyll degradation. The application of exogenous ABA was able to up-regulate many genes in relation to the carotenoids accumulation and chlorophyll breakdown, thus promoting the color transition of tomato fruit. ABA has also the potential to enhance the transcription of the genes related to antioxidant capacity, such as *SOD*s, *CAT*s, *APX*s, *GST*s, *GPX*s, *TrX*s and *PrxR*s etc. Besides, the elevated expression of genes involved in flavonoids biosynthesis after ABA exposure was striking, suggesting ABA could enhance the defense response by producing more secondary metabolite in tomato fruit. The present study is our preliminary analysis of ABA influence in ripening-related processes at transcriptome level. It could be assumed that the influences of ABA on these biological processes were possibly via multi-hormonal interactions in the regulation of fruit ripening. Therefore, these preliminary results from RNA-seq shed light for our further investigation on the regulatory mechanism of hormones crosstalk in fruit ripening at biological and molecular level. Moreover, the role of photosynthesis in ripening process of postharvest tomato is also an interesting topic worthy of further investigation.

## Supporting Information

S1 Fig Function Classification and KEGG Analysis of all expressed genes. (a) Gene Ontology (GO) functional annotation of genes. (b) Function classification in cluster of orthologous groups for eukaryotic complete genomes (KOG). (c) KEGG biochemical mappings for tomato fruit.
(TIF)

S2 Fig Analysis of all DEGs across the three samples. (a) A Venn diagram showing the number of commonly and specially expressed genes among ABA, NDGA and CK samples. (b) A histogram indicating the number of DEGs (an absolute value of log2 ratio≥1 and P value ≤0.05). The red columns represent the up-regulated DEGs and green columns represent the down-regulated DEGs.
(TIF)

S3 Fig Comparison between the fold changes in gene expression gained by qRT-PCR and RNA-seq analysis. Blue bar represented the result of RNA-seq and the red bar represented the result of qRT-PCR.
(TIF)

S4 Fig Correlation analysis of log_2_ fold change data generated by real time RT-PCR with that from RNA-seq. Sixteen genes with different expression patterns were selected for real time qRT-PCR analysis. The RNA-seq log2-fold change (X-axis) were plotted against the log2-fold change obtained by qRT-PCR (Y-axis). ABA versus CK **(a)**. NDGA versus CK **(b)**.
(TIF)

S1 Table Statistics of RNA-seq alignment. (XLS)

S2 Table The genes and transcripts generated in tomato transcriptome. (XLSX)

S3 Table The number of genes mapped to each GO term. (XLS)

S4 Table The number of genes mapped to each KEGG pathway. (XLS)

S5 Table The genes expressed in CK, ABA and NDGA treated samples. The different expression was analyzed according to the expression level of each gene among three samples by using P-value. yes: significantly difference expression (P value ≤ 0.05); no: no change in expression level (P value>0.05).
(XLS)

S6 Table Distribution of expression levels in CK, ABA and NDGA libraries. The genes’ expression level were normalized with the value in FPKM.
(XLS)

S7 Table Identification of highly enriched GO terms of DEGs. P value < 0.05, BP represents biological process, CC represents cellular component and MF represents molecular function.
(XLS)

S8 Table GO enrichment analysis of DEGs in response to exogenous ABA. (XLS)

S9 Table GO enrichment analysis of DEGs in response to exogenous NDGA. (XLS)

S10 Table Identification of highly enriched KEGG classes of DEGs (P value < 0.05). (XLS)

S11 Table KEGG pathway enrichment analysis of DEGs in response to exogenous ABA. (XLS)

S12 Table KEGG pathway enrichment analysis of DEGs in response to exogenous NDGA. (XLS)

S13 Table Genes related to carotenoid biosynthesis. The genes’ expression level were normalized with the value in FPKM. The different expression was analyzed according to the expression level of each gene among three samples by using P-value. yes: significantly difference expression (P value ≤ 0.05); no: no change in expression level (P value>0.05).
(XLS)

S14 Table Genes related to chorophyll degradation. The genes’ expression level were normalized with the value in FPKM. The different expression was analyzed according to the expression level of each gene among three samples by using P-value. yes: significantly difference expression (P value ≤ 0.05); no: no change in expression level (P value>0.05).
(XLS)

S15 Table Genes related to flavonoid biosynthesis. The genes’ expression level were normalized with the value in FPKM. The different expression was analyzed according to the expression level of each gene among three samples by using P-value. yes: significantly difference expression (P value ≤ 0.05); no: no change in expression level (P value>0.05).
(XLS)

S16 Table Genes related to reactive oxygen species (ROS) scavenging system. The genes’ expression level were normalized with the value in FPKM. The different expression was analyzed according to the expression level of each gene among three samples by using P-value. yes: significantly difference expression (P value ≤ 0.05); no: no change in expression level (P value>0.05).
(XLS)

S17 Table Genes related to photosynthesis pathway. The genes’ expression level were normalized with the value in FPKM. The different expression was analyzed according to the expression level of each gene among three samples by using P-value. yes: significantly difference expression (P value ≤ 0.05); no: no change in expression level (P value>0.05).
(XLS)

S18 Table Comparison between the results of qRT-PCR and RNA-seq. The relative expression data presented in the table are means ± SE of three biological replicates.
(XLS)

S19 Table The full name of 47 genes’ abbreviations presented in the manuscript. (DOC)
