# Supplementary figures and images for: Transcriptomic Analysis Reveals Possible Influences of ABA on Secondary Metabolism of Pigments, Flavonoids and Antioxidants in Tomato Fruit during Ripening
Source: PLoS One. 2015 Jun 8;10(6):e0129598. doi: 10.1371/journal.pone.0129598 (PMC4460000; doi:10.1371/journal.pone.0129598)

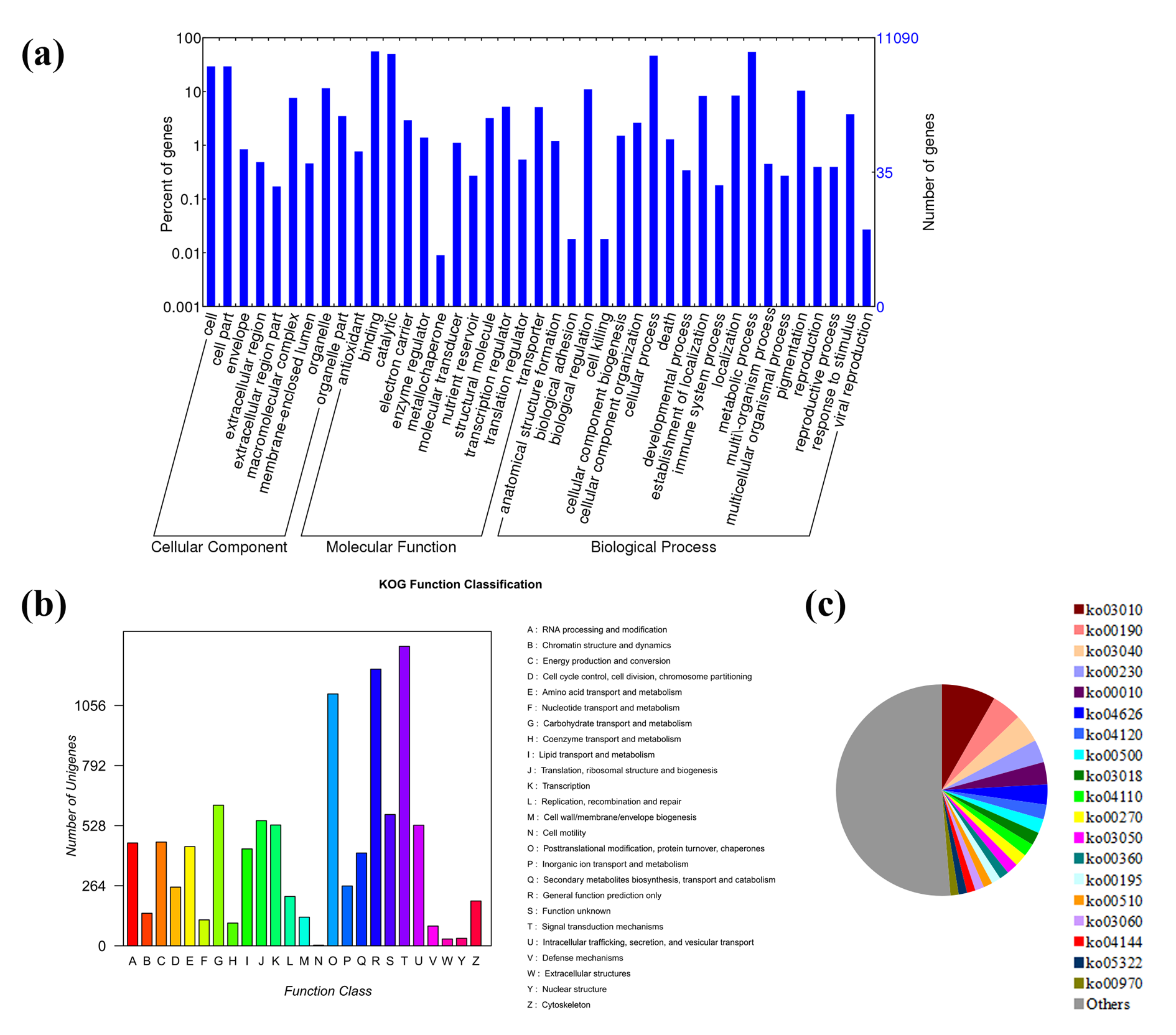

Supplement: S1 Fig — (a) Gene Ontology (GO) functional annotation of genes. (b) Function classification in cluster of orthologous groups for eukaryotic complete genomes (KOG). (c) KEGG biochemical mappings for tomato fruit. (TIF) [file pone.0129598.s001.tif]

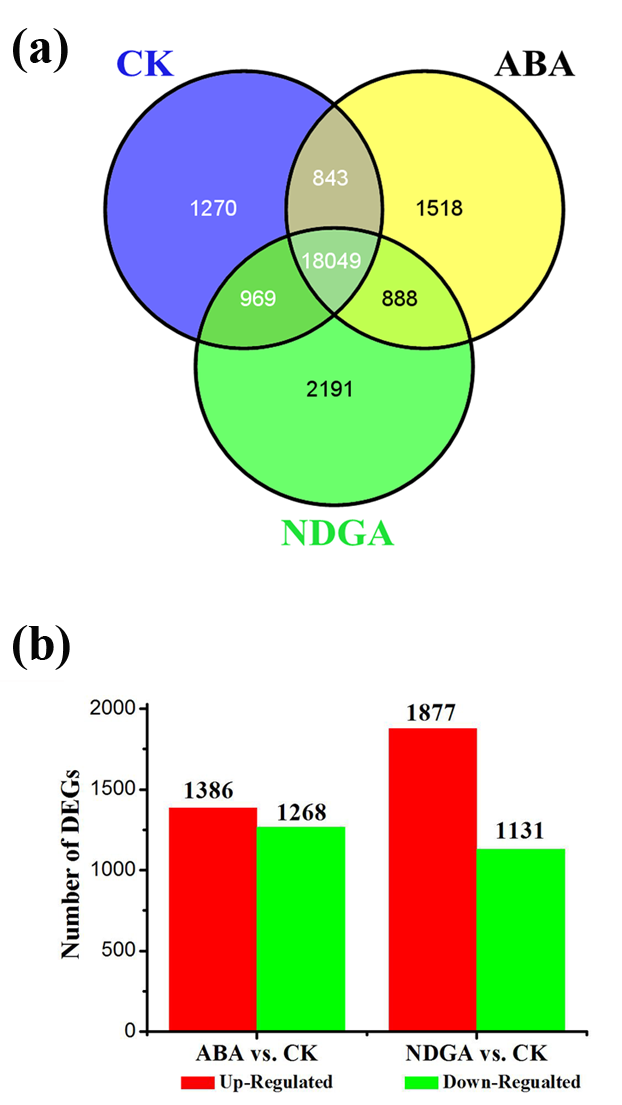

Supplement: S2 Fig — (a) A Venn diagram showing the number of commonly and specially expressed genes among ABA, NDGA and CK samples. (b) A histogram indicating the number of DEGs (an absolute value of log2 ratio≥1 and P value ≤0.05). The red columns represent the up-regulated DEGs and green columns represent the down-regulated DEGs. (TIF) [file pone.0129598.s002.tif]

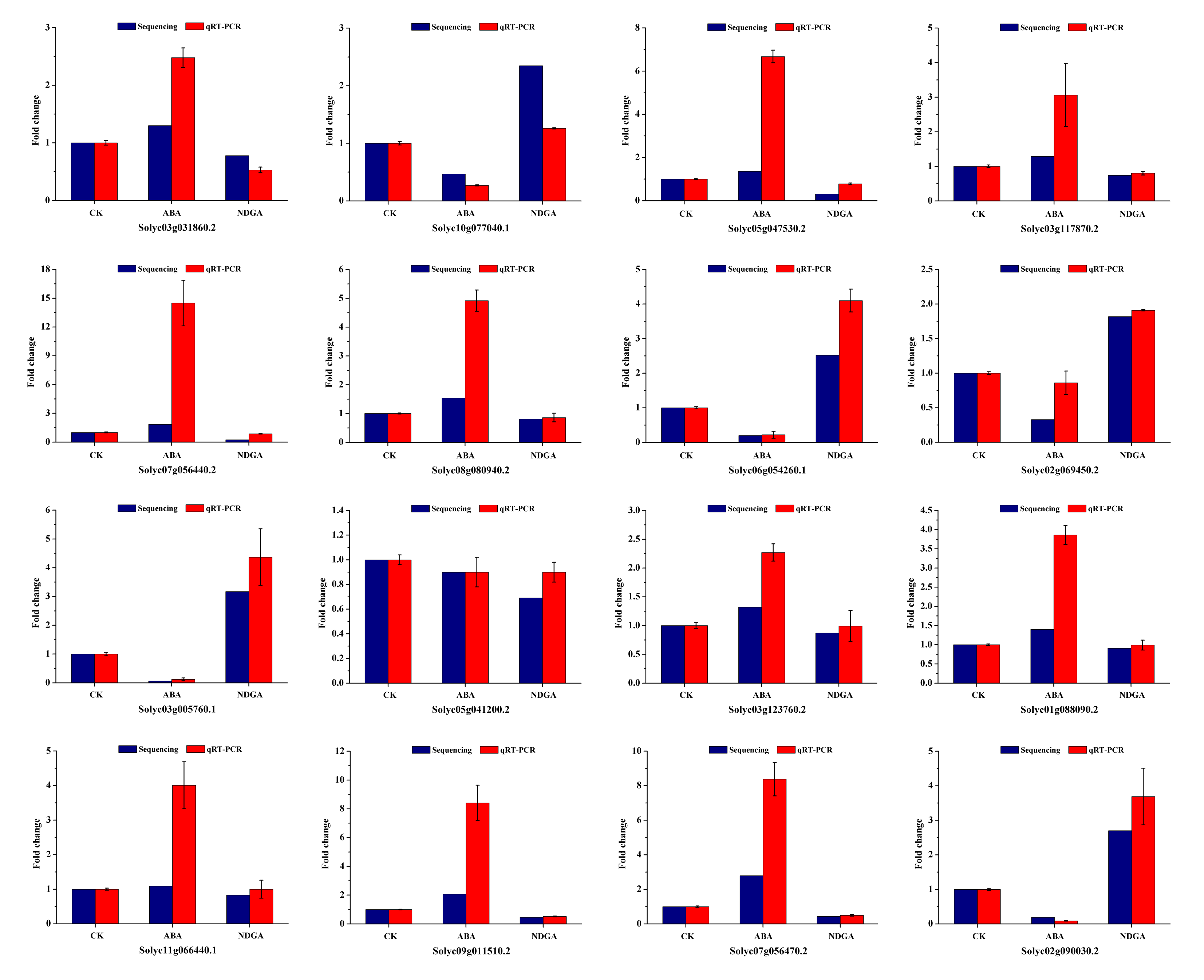

Supplement: S3 Fig — Blue bar represented the result of RNA-seq and the red bar represented the result of qRT-PCR. (TIF) [file pone.0129598.s003.tif]

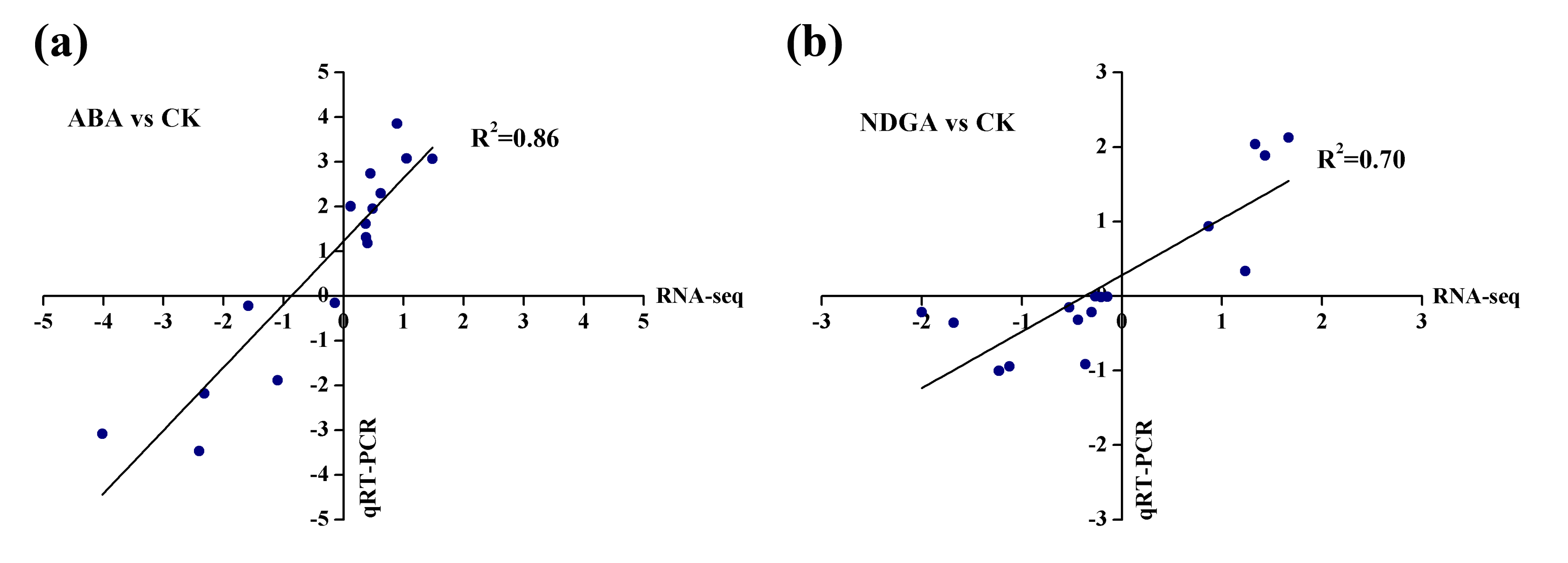

Supplement: S4 Fig — Sixteen genes with different expression patterns were selected for real time qRT-PCR analysis. The RNA-seq log2-fold change (X-axis) were plotted against the log2-fold change obtained by qRT-PCR (Y-axis). ABA versus CK (a). NDGA versus CK (b). (TIF) [file pone.0129598.s004.tif]
