# Supplementary material for: Transcriptomic Analysis Reveals Possible Influences of ABA on Secondary Metabolism of Pigments, Flavonoids and Antioxidants in Tomato Fruit during Ripening
Source: PLoS One. 2015 Jun 8;10(6):e0129598. doi: 10.1371/journal.pone.0129598 (PMC4460000; doi:10.1371/journal.pone.0129598)
Supplement: S19 Table — (DOC) [file pone.0129598.s023.doc]

**Table S19. The full name of 47 genes’ abbreviations presented in the manuscript.**

| **Abbreviations** | **Full name** |
| --- | --- |
| 4CL | 4-coumarate-CoA ligase |
| ACO | ACC oxidase |
| ACS | ACC synthetase |
| AGL8 | AGAMOUS-like 8 |
| APX | ascorbate peroxidase |
| C4H | cinnamate 4-hydroxylase |
| CAT | catalase |
| CHI | chalcone isomerase |
| Chlase | chlorophyllase |
| CHS | chalcone synthase |
| CRTISO | carotenoid isomerase |
| DHAR | carotenoid isomerase |
| DMAPP | dimethylallyl disphosphate |
| DXR | 1-deoxy-D-xylulose5-phosphate reductoisomerase |
| DXS | deoxyxylulose-5-phosphate synthase |
| F3H | flavanone 3-hydroxylase |
| FBA | fructose-bisphosphate aldolase 2 |
| FBP | fructose-1-6-bisphosphatase |
| FLS | flavonol synthase |
| GGPP | geranylgeranyl pyrophosphate |
| GGPS | geranylgeranyl pyrophosphate synthase |
| GLR | glutaredoxin |
| GPX | glutathione peroxdase |
| GR | glutathione reductase |
| GSH-ASA | glutathione-ascorbate |
| HCT | hydroxycinnamoyl-CoA shikimate/quinate hydroxycinnamoyl transferase |
| IPI | IPP isomerase |
| IPP | isopentenyl diphosphate |
| LHCA | photosystem I light harvesting complex gene |
| LHCB | photosystem II light harvesting complex gene |
| LYC | lycopene cyclase |
| MDAR | monodehydroascorbate reductase |
| NSY | neoxanthin synthase |
| PAL | phenylalanine ammonia-lyase |
| PAP1 | Probable plastid-lipid-associated protein 1 |
| PaO | pheide a oxygenase |
| PDS | phytoene desaturase |
| PPH | pheophytinase |
| PrxR/TrX | peroxiredoxin/thioredoxin |
| PSY | phytoene synthetase |
| RBCB | ribulose bisphosphate carboxylase small chain |
| RCCR | red Chl catabolite reductase |
| RUBPCO | ribulose 1,5-bisphosphate carboxylase |
| SOD | superoxide dismutase |
| UFGT | UDP flavonoid glucosyltransferase |
| ZDS | zeta-carotene desaturase |
| ZEP | zeaxanthin epoxidase |
